# Nitric oxide is required for an optimal establishment of the *Medicago truncatula–Sinorhizobium meliloti* symbiosis

**DOI:** 10.1111/j.1469-8137.2011.03693.x

**Published:** 2011-07

**Authors:** Jennifer del Giudice, Yvan Cam, Isabelle Damiani, Franck Fung-Chat, Eliane Meilhoc, Claude Bruand, Renaud Brouquisse, Alain Puppo, Alexandre Boscari

**Affiliations:** 1 UMR INRA 1301/CNRS 6243/Université de Nice – Sophia Antipolis, Interactions Biotiques et Santé Végétale, Institut Agrobiotech400 route des Chappes, BP 167, F–06903 Sophia-Antipolis Cedex, France; 2 UMR CNRS 2594/INRA 441, Laboratoire des Interactions Plantes MicroorganismesF–31320 Castanet Tolosan, France

**Keywords:** flavohaemoglobin, MtCRE1, nitric oxide, nitrogen fixation, nodule initiation and organogenesis, *Rhizobium* legumes symbiosis

## Abstract

- Nitric oxide (NO) is a gaseous molecule that participates in numerous plant signalling pathways. It is involved in plant responses to pathogens and development processes such as seed germination, flowering and stomatal closure.
- Using a permeable NO-specific fluorescent probe and a bacterial reporter strain expressing the *lacZ* gene under the control of a NO-responsive promoter, we detected NO production in the first steps, during infection threads growth, of the *Medicago truncatula*–*Sinorhizobium meliloti* symbiotic interaction. Nitric oxide was also detected, by confocal microscopy, in nodule primordia.
- Depletion of NO caused by cPTIO (2-(4-carboxyphenyl)-4,4,5,5-tetramethyl imidazoline-1-oxyl-3-oxide), an NO scavenger, resulted in a significant delay in nodule appearance. The overexpression of a bacterial *hmp* gene, encoding a flavohaemoglobin able to scavenge NO, under the control of a nodule-specific promoter (pENOD20) in transgenic roots, led to the same phenotype. The NO scavenging resulting from these approaches provoked the downregulation of plant genes involved in nodule development, such as *MtCRE1* and *MtCCS52A*. Furthermore, an Hmp-overexpressing *S. meliloti* mutant strain was found to be less competitive than the wild type in the nodulation process.
- Taken together, these results indicate that NO is required for an optimal establishment of the *M. truncatula–S. meliloti* symbiotic interaction.

## Introduction

Nitric oxide (NO) is a gaseous signalling molecule that has been established as a major signal in mammals (Ignarro, 2000). The participation of NO in a large number of plant signalling pathways is now well established (Grun *et al.*, 2006) and there is increasing evidence of its role in plant growth and development (del Rio *et al.*, 2004; Delledonne, 2005). Its involvement in seed dormancy (Bethke *et al.*, 2006), germination and hypocotyl elongation (Beligni & Lamattina, 2000), flowering (He *et al.*, 2004) and regulation of stomatal closure (Neill *et al.*, 2002) has also been demonstrated, although the pathway of its synthesis in plants is still a matter of debate (Moreau *et al.*, 2008).

The importance of NO in plants emerged from pioneering works on plant responses to pathogens (Delledonne *et al.*, 1998; Durner *et al.*, 1998). Nitric oxide signalling in the induction of cell death, defence genes and interaction with reactive oxygen species (ROS) during plant defence against pathogen attack is well documented (Neill *et al.*, 2003; Delledonne, 2005; Mur *et al.*, 2006; Besson-Bard *et al.*, 2008). Nitric oxide not only plays an important role in the plant hypersensitive response (HR), a localized programmed cell death (PCD) that confines the pathogen to the site of attempted infection, but also in non PCD defence (Mur *et al.*, 2006). Conversely, there is also evidence of its potential importance within pathogens. Detoxification of NO in bacteria has been intensively investigated. Under aerobic conditions, it occurs through the nitric oxide dioxygenase activity of the flavohaemoglobin, which converts it to nitrate (Gardner *et al.*, 1998; Poole & Hughes, 2000). Bacterial mutants lacking flavohaemoglobin are hypersensitive to NO (Poole & Hughes, 2000) and flavohaemoglobins are important for animal pathogens to survive host-derived NO (Stevanin *et al.*, 2002; Gilberthorpe *et al.*, 2007; Svensson *et al.*, 2010). Examining the genomes of several bacterial phytopathogens has revealed the conservation of the *hmp* gene (Mur *et al.*, 2006). Furthermore, mutation of the *hmpX* gene from *Dickeya dadantii* (formerly *Erwinia chrysantemi*) resulted in a significant increase in NO concentrations and led to a resistance response of the HR type. Conversely, expression of *hmpX* into *Pseudomonas syringae* pv. *tomato avrB* suppressed the HR elicited in Arabidopsis (Boccara *et al.*, 2005). Such data demonstrate that the scavenging of NO by certain pathogens is a requirement for successful infection.

Contrary to pathogenic situations, the interaction between legumes and soil bacteria of the Rhizobiaceae leads, after extensive recognition by both partners, to the establishment of a symbiotic relationship characterized by the formation of new differentiated organs called nodules, which provide a niche for bacterial nitrogen fixation. Functional nodules result from the combination of developmental and infectious processes; bacteria released in plant differentiate into bacteroids with the unique ability to fix atmospheric nitrogen via nitrogenase activity (Long, 2001; Oldroyd & Downie, 2008).

Several studies have demonstrated the occurrence of NO production during legume–*Rhizobium* symbiosis. A nitric oxide synthase-like activity has been measured in lupine nodules (Cueto *et al.*, 1996). Nitric oxide complexed to leghaemoglobin (Lb), the haemoprotein ensuring O_2_ fluxes to bacteroids, has been detected in soybean nodules using electron paramagnetic resonance (EPR) (Mathieu *et al.*, 1998; Meakin *et al.*, 2007). This nitrosylleghaemoglobin (LbNO) was mainly observed in young nodules, which led the authors to propose a relationship between LbNO abundance and nodule development and/or functioning (Mathieu *et al.*, 1998). Production of NO during the early steps of legume–rhizobia symbiosis has also been reported by Shimoda *et al.* (2005) and NO, together with auxin, has been shown to control indeterminate nodule formation (Pii *et al.*, 2007). In the same way, gene expression of non-symbiotic haemoglobin (Hb) in *Lotus japonicus* has been shown to be transiently induced by the inoculation with the symbiotic bacteria and by an NO donor (Shimoda *et al.*, 2005; Nagata *et al.*, 2008). Although the functions of class 1 Hbs are not fully understood, they appear to play some role in relation to NO metabolism (Igamberdiev & Hill, 2004) and in the establishment of the symbiosis (Nagata *et al.*, 2008). Furthermore, modulation of the NO level by overexpression of class 1 plant Hb genes appeared to enhance symbiotic nitrogen fixation activity between *L. japonicus* and *Mesorhizobium loti* (Shimoda *et al.*, 2009). Based on the use of a permeable NO-sensitive fluorescent probe, NO formation has been detected in bacteroid-containing cells of the nodule fixation zone in *Medicago truncatula–Sinorhizobium meliloti* functional nodules (Baudouin *et al.*, 2006). A wide modulation of NO-responsive genes has been detected during the establishment of a *M. truncatula–S. meliloti* functioning nodule, pointing to a possible contribution of NO to nodule metabolism (Ferrarini *et al.*, 2008). Moreover, the response to NO of *S. meliloti* has been studied recently via a transcriptomic approach. Among genes responding to NO, was a flavohaemoglobin-encoding gene. An *hmp* mutant displayed a higher sensitivity toward NO in culture and led to reduced nitrogen fixation efficiency *in planta* (Meilhoc *et al.*, 2010). Together, these data indicate that NO is produced at different steps of the symbiotic process, where it may play different roles.

In this work, using pharmacological and genetic approaches, we investigated the presence of NO and its possible role(s) in the first steps of the *M. truncatula–S. meliloti* symbiosis. Our data suggest that NO is required for an optimal establishment of the symbiotic interaction.

## Materials and Methods

### Bacterial strains and growth conditions

Bacterial strains and plasmids are listed in Table 1. *Escherichia coli* strains were propagated in Luria–Bertani (LB) medium. *Sinorhizobium meliloti* strains were constructed and grown in LB medium supplemented with 2.5 mM CaCl_2_ and 2.5 mM MgSO_4_ (LBMC). Antibiotics, when required, were added at the following concentrations: streptomycin 100–300 μg ml^−1^, tetracycline 5–10 μg ml^−1^, gentamycin 40 μg ml^−1^, carbenicillin 50 μg ml^−1^, kanamycin 50 μg ml^−1^.

**Table 1:** Strains and plasmids used in this study

Strain or plasmid	Description	Reference or source
*Sinorhizobium meliloti*		
GMI11495	wt strain, SU-47 derived, Sm^R^	Pobigaylo *et al.* (2008)
RCR2011	wt strain, SU-47 derived	Rosenberg *et al.* (1981)
RCR2011-DsRed	RCR2011 containing pDG77	E. Andrio, unpublished
GMI11549	GMI11495 *hmp* :: mTn5-STM-2.12.A01	A. Becker, see Pobigaylo *et al.* (2008)
CBT515	Rm1021 *nnrR* :: pVO155	Meilhoc *et al.* (2010)
GMI11545	GMI11495 containing pXLGD4	D. Capela, unpublished
CBT602	GMI11495 containing pBBR-hmp	Meilhoc *et al.* (2010)
*Escherichia coli*		
DH5α	*supE44*Δ*lac*U169 (Φ80 *lacZ*ΔM15)	Sambrook *et al.* (1989)
	*hsdR17 recA1 endA1 gyrA96 thi-1 relA1*	
Plasmids		
pRK2013	Helper plasmid for triparental matings	Figurski & Helinski (1979)
pXLGD4		Ditta *et al.* (1985)
pGEM-T	Cloning vector, Amp^R^	Promega
pBBR1MCS-5	Cloning vector, Gm^R^ derivative of pBBR1	Kovach *et al.* (1995)
pVO155	Integrational plasmid with promoterless *uidA* gene, Kan^R^	Oke & Long (1999)
pBBR-hmp	pBBR1MCS-5 + *hmp*	Meilhoc *et al.* (2010)
pDG77	Expresses DsRed under *Salmonella typhimurium pTrp* promoter	Bringhurst *et al.* (2001)
pCZ962	Derived from pCZ917 cloning vector by insertion of a terminator upstream from *lac*Z Tc^R^ Amp^R^	C. Zischek *et al.*, unpublished
psma1289-lacZ	*sma1289* promoter in pCZ962 cloning vector with promoterless lacZ gene	This work

To test the bacterial response to NO, *S. meliloti* was grown in Vincent minimal medium (VMM: 7.35 mM KH_2_PO_4_, 5.74 mM K_2_HPO_4_, 1 mM MgSO4, 18.7 mM NH_4_Cl, 10 mM Na_2_ succinate, 456 μM CaCl_2_, 35 μM FeCl_3_, 4 μM biotin, 48.5 μM H_3_BO_3_, 10 μM MnSO_4_, 1 μM ZnSO_4_, 0.5 μM CuSO_4_, 0.27 μM CoCl_2_, 0.5 μM NaMoO_4_, pH = 7) at 28°C. The NO donor Spermine NONOate (SpNN) was purchased from Cayman Chemicals Coger (CAY-82150-M100, Paris, France). A 100 mM stock solution was prepared, just before use, in Na-phosphate buffer (0.1 M) pH 6.9 and then diluted in the cell culture medium at the appropriate concentration.

### Plants and growth conditions

**Fig. 1:**
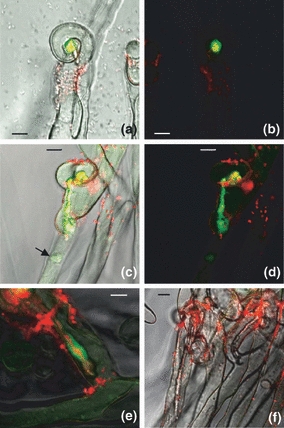
Detection of nitric oxide (NO) during infection threads growth by confocal laser scanning microscopy. The green fluorescence of the DAF-2DA was observed during root hair infection in the ‘shepherd's crooks’ (a–b) and during infection threads progression (c–f). Plants were inoculated with a *Sinorhizobium meliloti* strain tagged with constitutive red fluorescent protein (RFP). (a), (c) and (f) were obtained from the merged pictures of the two fluorescents markers over the bright field. (b) and (d) correspond to (a) and (c) without bright field; (e) shows an higher magnification of IT. The plants in (f) were pretreated with 1 mM of cPTIO (2-(4-carboxyphenyl)-4,4,5,5-tetramethyl imidazoline-1-oxyl-3-oxide) for 45 min. The arrow on (c) indicates the nucleus. In the acquisition settings used for the experiment, autofluorescence of the infection thread was negligible. Furthermore, the cPTIO treatment significantly reduced the fluorescence (f). The typical fluorescence emission spectra of the DAF probe was also confirmed (Data not shown). Data are from five independent experiments (*n*=43) and pictures shown are representative of the different conditions. Bars, (a–d,f) 10 μm, (e) 5 μm.

*Medicago truncatula* cv Jemalong A17 was used, and was cultivated as follows. Seeds of *M. truncatula* cv Jemalong A17 were scarified with H_2_SO_4_, surface-sterilized in a bleach solution, rinsed with sterile distilled water, germinated on agar plates in the dark and allowed to grow on nitrogen-free Farhäeus medium in test tubes or plates (covered with pouch paper) for 2 to 7 d before inoculation in a culture room (22–25°C) with a 16 h light/8 h dark photocycle (all details and medium used are described in the Medicago Handbook: http://www.noble.org/MedicagoHandbook/). For NO detection with 4,5-diaminofluorescein (DAF-2, Sigma-Aldrich) (Figs 1, 4), transgenic roots (Fig. 6), and quantitative reverse-transcription polymerase chain reaction (qRT-PCR) experiments (Fig. 7), *S. meliloti* strains were grown in liquid LBMC medium, cells were pelleted at 2500 ***g***, washed twice with sterile distilled water, resuspended in sterile distilled water to a final optical density at 600 nm (OD_600_) of 0.01, and plants were inoculated with 200 μl of bacterial suspension per root. In all other experiments, *S. meliloti* strains were grown on LBMC plates, cells were resuspended in sterile distilled water to a final OD_600_ of 0.001, and plants were inoculated with 100 μl of bacterial suspension per root. When 2-(4-carboxyphenyl)-4,4,5,5-tetramethyl imidazoline-1-oxyl-3-oxide (cPTIO; Sigma) was used, 200 μl of a 1 mM solution prepared in sterile water was added along the whole length of the roots, 2 h before, and 2 h after inoculation, and then, every 24 h during 4 d.

**Fig. 2:**
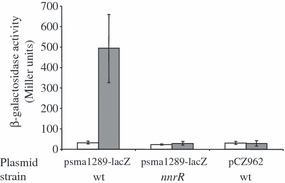
Expression of the sma1289-lacZ fusion in *Sinorhizobium meliloti* cultures. Wild type (wt) (GMI11495) or *nnrR* (CBT515) *S. meliloti* strains carrying the sma1289–lacZ fusion, and the wt strain harbouring the empty plasmid (pCZ962) were grown to exponential phase (OD_600_ 0.2). β-Galactosidase activity was measured on cultures grown 1.5 h without (open bars) or with (tinted bars) 25 μM spermine NONOate. Three independent experiments were performed. The mean (± mean deviation) is shown on the graph.

**Fig. 3:**
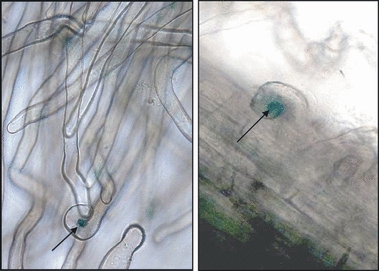
Nitric oxide (NO) detection in *Medicago truncatula* root hairs using the nitric oxide (NO) biosensor *Sinorhizobium meliloti* strain. The *M. truncatula* roots were inoculated with the wild-type (wt) *S. meliloti* strain harbouring the sma1289–lacZ fusion. Roots were stained for 12 h for β-galactosidase activity detection. Observations were made with an optical microscope (200×). Approximately 25 roots were observed between 4 d and 6 d after inoculation. Two representative images are shown. We typically observed two or three stained curls per root (arrows), a number consistent with the number of infection events (four of five per root, as estimated using GMI11545, a wt strain constitutively expressing *lacZ*).

**Fig. 4:**
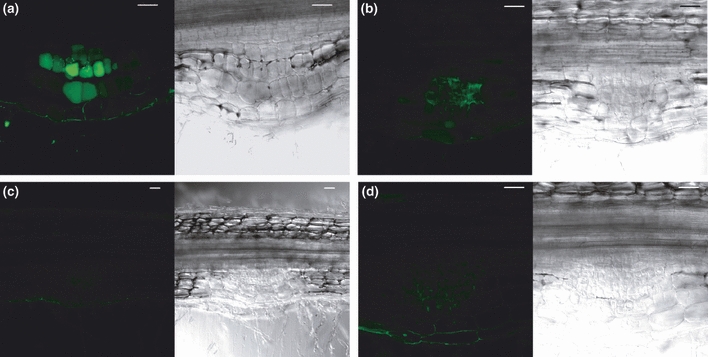
Detection of nitric oxide (NO) in nodule primordium by confocal laser scanning microscopy. DAF-2DA fluorescence (green) was observed on fresh slices (100 μm) of the root infection zone. The roots slices on (b) and (d) were pretreated with 1 mM of cPTIO (2-(4-carboxyphenyl)-4,4,5,5-tetramethyl imidazoline-1-oxyl-3-oxide) for 45 min before the treatment with the DAF-2DA probe. Each picture is composed by the DAF fluorescence and transmission micrographs. In pictures (a) and (b), DAF fluorescence was observed in dividing cells of the nodule primordium. The cPTIO treatment reduced significantly the fluorescence (c,d) which confirmed the specificity of the fluorescence. Root slices analysed without incubation with the DAF-2DA probe did not show autofluorescence in our acquisition settings. The typical fluorescence emission spectrum of the DAF probe was also confirmed (Data not shown). Data are from four independent experiments (*n*=48) and pictures shown are representative of the different conditions. Bars, 50 μm.

**Fig. 6:**
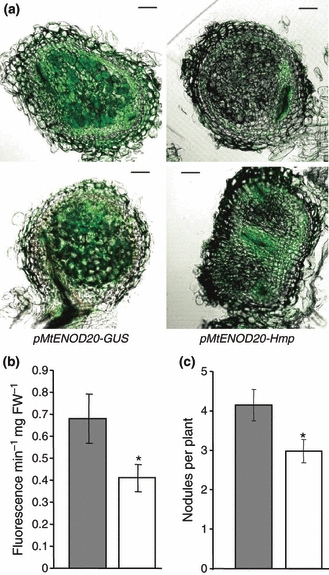
Nitric oxide (NO) measurement and nodulation phenotype on transgenic hairy roots overexpressing the *Sinorhizobium meliloti* Hmp flavohemoglobin under the control of the nodule specific promoter pMtENOD20. (a) Representative confocal microscopic pictures obtained from observation with DAF-2DA probe of transgenic Hmp-overexpressing and control glucuronidase (GUS) nodules slices (11 d post-inoculation). Bar, 100 μm. (b) Quantification of the NO levels by spectrofluorimetric method. The Hmp-overexpressing and control (GUS) transgenic roots were incubated with nonpermeant DAF2 probe to measure the NO produced and released in the medium. Results presented from roots 5 dpi. *n*=68 and *n*=62 for pMtENOD20-GUS (tinted bar) and pMtENOD20-hmp (open bar), respectively. Asterisk indicates a statistically significant difference (Student's *t*-test, *P*<0.05). (c) Number of nodules induced by the wild-type *S. meliloti* strain on transgenic Hmp-overexpressing and control hairy roots measured 11 d post-inoculation. Three independent experiments were done with a number of plants, *n*=78 for pMtENOD20-GUS (tinted bar), and *n*=69 for pMtENOD20-hmp (open bar). Asterisk indicates a statistically significant difference (Student's *t*-test, *P*<0.05).

**Fig. 7:**
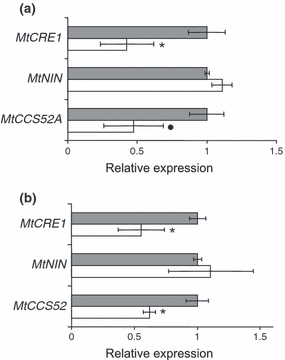
Effect of nitric oxide (NO) modulation on transcripts level of genes involved in symbiotic interaction. (a) Real-time reverse-transcription polymerase chain reaction (RT-PCR) analysis of *MtCRE1*, *MtNIN* and *MtCCS52A* genes on inoculated roots (4 d post-inoculation) treated with (open bars) or without (tinted bar) 1 mM of cPTIO (2-(4-carboxyphenyl)-4,4,5,5-tetramethyl imidazoline-1-oxyl-3-oxidec) during 8 h. (b) Real-time RT-PCR analysis of *MtCRE1*, *MtNIN* and *MtCCS52* genes on *hmp*-overexpressing (open bars) and control (tinted bars) transgenic hairy roots 11 d post-inoculation. For these two analyses, at least three independent experiments were performed. Asterisk indicates a statistically significant difference (Student's *t*-test, *P*<0.05) compared with the control. A dot indicates a statistically significant difference between *P*<0.05 and *P*<0.1 (Student's *t*-test) compared with the control.

For competition assays, plants were inoculated with a 1 : 1 mixture of wild-type (wt) and mutant strains (OD_600_ = 0.001). In order to verify that the number of cells used for inoculation with each strain were identical, CFUs were determined for each strain. To determine the relative proportions of nodules occupied by mutant or wt bacteria, the wt strain contained a plasmid (pXLGD4) constitutively expressing the β-galactosidase enzyme. After 3 wk, nodules were wounded and incubated (1 h) in Z′ buffer (0.1 M Na_2_HPO_4_/NaH_2_PO_4_ pH 7.4, 10 mM KCl, 1 mM MgSO_4_) containing 1.5% of glutaraldehyde under vacuum. Nodules were then washed three times in Z′ buffer and incubated in the same buffer containing potassium ferricyanide (5 mM) and X-gal (0.8% in dimethylformamide). Incubation was performed under vacuum at room temperature (90 min) and then at 37°C (90 min). Blue-stained nodules corresponding to nodules infected by GMI11545 (wt) and pink nodules corresponding to nodules infected by the mutant strain were counted. For each competition test, 50 –100 nodules from 10–20 plants were analysed, and the mean value of the percentage of nodule occupancy was calculated for each strain. When cPTIO was used in the test, nodules were taken at 4 wk instead of 3 wk after inoculation because of the delayed nodulation.

### Plasmid constructions

The binary vector pKm43GW-rolD::GFP (Van de Velde *et al.*, 2006) was modified by replacing the green fluorescent protein (GFP) with a red fluorescent protein (RFP) reporter cassette under the control of the rolD promoter to facilitate the analysis by confocal microscopy with the cell permeable 4,5-Diaminofluorescein diacetate (DAF-2DA) fluorescent probe (excitation 495 nm, emission max *c.* 515 nm; Sigma-Aldrich) and the selection of transgenic roots. The RFP cassette was amplified by PCR from the pK7RWG2 vector (http://gateway.psb.ugent.be) with the primers DsRedX and DsRedS containing, respectively, a *Xho*I and *Sac*I restriction site and cloned in the pKm43GW-rolD by using these two restriction sites to get the pKm43GW-rolD::RFP.

The promoter from *MtENOD20* (Accession: AC136953.17; GenBank accession no. X99467) (2050 bp) was amplified with the specific primers a74F and a74R containing the corresponding attB recombination sites. The promoter region was recombined into pDONR-P4-P1 (Invitrogen). The Multisite Gateway Three-Fragment Vector Construct kit (Invitrogen) was used to fuse the promoter region with the *GUS* gene (from pDONR207-GUS) or the *hmp* gene (from pDONR207-*hmp*) and the T35S terminator (from pENTR-R2-T35S-L3) in the pKm43GW-rolD-RFP vector. The *hmp* gene was amplified from *S. meliloti* genomic DNA with the specific primers a76F and a76R.

The bacterial NO biosensor was constructed as follows. The promoter of the *S. meliloti* gene *sma1289* was amplified by PCR with the following primers: OCB744 and OCB745 containing the *Pst*I and *Xba*I restriction sites, respectively. The PCR amplification was performed using genomic DNA of strain GMI11495 as a template. The amplified fragment (410 nucleotides) was ligated in pGEM-T (Promega). The cloned *S. meliloti* DNA region was verified by DNA sequencing. The pGEM-T plasmid containing the *sma1289* promoter was then digested with *Xba*I and *Pst*I, and ligated into the *Xba*I and *Pst*I digested pCZ962 plasmid containing the promoterless *lacZ* gene. Plasmids (pCZ962 with or without the *sma1289* promoter) were introduced in *S. meliloti* strains (GMI11495, CBT515, CBT602) by tri-parental mating using pRK2013 as a helper, and subsequent selection for antibiotic resistance.

### β-galactosidase activity assay

*Sinorhizobium meliloti* cells were grown to exponential phase at 28°C in VMM. At OD_600_ = 0.2, Spermine NONOate (0 or 25 μM) was added to the cultures which were then incubated at 28°C. After 1.5 h incubation, OD_600_ was measured and aliquots (1 ml) of the cultures were centrifuged. The pellets were immediately frozen in liquid nitrogen and stored at −20°C. β-Galactosidase assays were performed on the thawed samples as described previously (Miller, 1972).

### β-galactosidase and β-glucuronidase detection *in planta*

For β-galactosidase detection, plants were inoculated with strains GMI11495 (wt), CBT515 (*nnrR*), or CBT602 (*hmp*^*++*^) carrying the plasmid pSma1289–lacZ or with strain GMI11495 carrying the empty plasmid. Six days after inoculation, plant roots were incubated (1 h) under vacuum, in Z′ buffer containing 1.5% glutaraldehyde. Roots were then washed three times in Z′ buffer and incubated (room temperature) in Z′ buffer containing potassium ferricyanide (5 mM) and X-gal (0.8% in dimethylformamide) for 90 min or for a longer period when the staining was too weak.

For β-glucuronidase detection, roots 5 d post-inoculation (dpi) and 13 dpi transformed with pKm43W-pENOD20-GUS were incubated for 1 h in acetone 90% (diluted in a phosphate buffer: Na_2_HPO_4_, NaH_2_PO_4_ 0.1M pH = 7.4) at −20°C. Roots were then washed twice in the phosphate buffer and incubated at room temperature, protected from light, in phosphate buffer containing potassium ferricyanide (0.5 mM) and X-gluc (0.5 ng ml^−1^) for 3 h and 16 h.

The roots were observed by optical microscopy (Axioplan imagin2; Zeiss). Pictures were taken with an AxioCam (Zeiss) and acquired with the corresponding software.

### *Agrobacterium rhizogenes* root transformation and inoculation

The construct pKm43GW-pENOD20-*hmp* was introduced into *Agrobacterium rhizogenes* strain *Arqua1* (Quandt *et al.*, 1993). *Medicago truncatula* plants were transformed with *Agrobacterium rhizogenes*, as described by Boisson-Dernier *et al.* (2001). Control plants were transformed with *A. rhizogenes* containing the pKm43GW-pENOD20-GUS. Selection of hairy roots took place 21 d after transformation. Plants were screened for transgenic roots by RFP with a MZFLII stereomicroscope (Leica, Wetzlar, Germany). One transgenic root per plant was retained, and the composite plants were transferred onto agar plates containing modified Fahräeus medium without nitrogen. Plants were inoculated 3 d after transfer.

### Measurement of NO production

The NO produced by both the wt and transgenic hairy roots, and released into the detection medium, was measured using the fluorescent probe DAF-2. At various times, the fluorescence of DAF-2T, the reaction product formed from DAF-2 and NO, was measured using a microplate reader spectrofluorimeter (Cary Eclipse; Varian, Les Ulis, France), excitation wavelength 495 nm/emission wavelength 515 nm. Plants were pooled into groups of seven to nine plants, and the roots were put into 3 ml of detection medium: 10 mM Tris-KCl detection buffer (DB: 10 mM Tris-HCl pH 7.4, 10 mM KCl) in the presence of 10 μM DAF-2 fluorescent probe. The roots were protected from light with tinfoil while the leaves were left in contact with air and light, allowing the photosynthesis to take place. Production of NO was measured at 10, 30, 60 and 90 min after the addition of the DAF-2 probe into the detection medium. Blank assays contained detection medium without plants.

### Confocal microscopy

Experiments were performed on young entire roots and fresh nodule slices (100 μm) obtained with a vibratome 1000 Plus (Labonord, Templemars, France). Plant material was then incubated for 30 min in the dark in 100–500 μl of DB containing 10 μM DAF-2DA the cell permeable analog of DAF-2 probe (Sigma-Aldrich). Plant tissues were subsequently washed for 30 min with DB and then mounted between slide and coverslip in DB for observation. When cPTIO was used, plant tissues were preincubated for 45 min in DB containing 1 mM of cPTIO, subsequently labelled with DAF-2DA and washed three times during 20 min with DB. The formation of DAF-2T following the NO reaction with DAF-2DA was visualized using a Zeiss LSM 500 confocal laser microscope upon excitation at 488 nm with an argon 2 laser. Dye emission was recorded using a 505–530 nm band-pass filter coupled with a 515 nm long-pass filter. Autofluorescence was judged negligible at all steps before acquisition with the DAF-2DA probe. Data acquisition settings (laser power, pinhole size, filters, detectors settings and scan conditions) were identical in all experiments. Images were processed and analysed using the Zeiss LSM510 meta software and Adobe Photoshop.

### Total RNA isolation, reverse transcription and gene expression analysis

For the cPTIO treatment used to identify NO-regulated genes, plantlets were grown on plates and then, at 4 d post-inoculation, were treated with 200 μl of 1 mM of cPTIO for 8 h. A control assay was not treated with cPTIO. Furthermore, in order to obtain a higher concentration of RNA from nodule primordia, a 2 cm region of the root in which most of the nodule primordia appear was harvested. To localize this root infection zone (IZ), we marked the position of the primary root apex on the day of the inoculation. Four days later, the IZ was mainly located around this mark.

Aproximately 100–200 mg of plant material (root) were ground in liquid nitrogen and total RNA was isolated using Trizol Reagent (Invitrogen). The integrity of total RNA was checked on agarose gels and its quantity as well as purity was determined spectrophotometrically. About 500–1500 ng of RNA was used as a template for reverse transcription reaction in 20 μl reaction volume using the Omniscript RT Kit (Qiagen). Quantitative real-time RT-PCR was carried out using the qPCR Mastermix Plus for SYBR Green I reagent (Eurogentec, http://www.eurogentec.com). Reactions were run on the Chromo4 Real-Time PCR Detection System (Bio-Rad) and quantification was performed with the Opticon Monitor analysis software v. 3.1 (Bio-Rad). Each reaction was set up in three technical replicates. For each reaction, 5 μl of 100-fold-diluted cDNA and 0.3 μM primers were used. The initial denaturing time was 10 min, followed by 40 PCR cycles at 95°C for 10 s, and 60°C for 1 min. The specificity of the amplification was confirmed by a single peak in a dissociation curve at the end of the PCR reaction. Data were quantified using Opticon Monitor 2 (MJ Research, Waltham, Massachusetts, USA) and analysed with RqPCRBase, an R package working on R computing environment for analysis of quantitative real-time PCR data (T. Tran & F. Hilliou, unpublished). The mRNA levels were normalized against three constitutively expressed endogenous genes, *Mtc27* (Mtr.27459.1.S1_s_at) (Van de Velde *et al.*, 2006) and two genes (Mtr.10324.1.S1_at; Mtr.31250.1.S1_at) selected as reference genes because of their stable expression profile in different microarray results (Benedito *et al.*, 2008). The PCR reactions for each biological replicate were performed in triplicate. For each experiment, the stability of the reference genes across samples was tested using genorm software (Vandesompele *et al.*, 2002). The absence of genomic DNA contaminations in the RNA samples was tested by PCR analysis of all samples using oligonucleotides bordering an intron in the glutathione synthetase gene of *M. truncatula.* The gene-specific primers used are listed in the Supporting Information, Table S1.

## Results

### NO is produced in infection threads and nodule primordia

To detect NO during the early steps of the *M. truncatula–S. meliloti* interaction, we used the cell-permeable NO-specific fluorescent probe, DAF2-DA (Kojima *et al.*, 1998). Between 2 dpi and 6 dpi, the formation of the fluorescent triazol derivative, DAF-2T, was visualized in infection threads (ITs). For all these experiments, roots were inoculated with a *S. meliloti* DsRed strain harbouring the pDG77 vector, which exhibits a constitutive red fluorescence. A yellow colour from the merging of the two fluorescent markers occurred (Fig. 1a–d) in the infection pockets, where rhizobia are enclosed within an apoplastic space created by root hair tip curling (the so-called ‘shepherd's crooks’) upstream of the IT. Interestingly, the red fluorescence from the bacteria and the green fluorescence from the DAF probe did not overlap along the ITs (Fig. 1c–e). On a higher magnification (Fig. 1e), the DsRed-labelled bacteria can be visualized in the centre of the IT surrounded by the green fluorescence. In Fig. 1(c) and (d), we can observe the position of the nucleus close to the IT tip indicating that the IT is growing as mentioned by Fournier *et al.* (2008). To validate the specificity of the green fluorescence observed, the roots were treated with the NO scavenger (cPTIO). The cPTIO treatment extinguished the fluorescence and further established that it was representative of NO production (Fig. 1f).

To confirm the presence of NO in the infection threads of *M. truncatula* root hairs, and to ascertain whether bacteria respond to the presence of NO in this environment, we constructed a reporter plasmid (psma1289-lacZ) carrying a transcriptional *lacZ* fusion to the *S. meliloti sma1289* gene promoter. The *sma1289* gene was chosen since it is known to be upregulated by NO via the NO-specific regulator NnrR (de Bruijn *et al.*, 2006; Meilhoc *et al.*, 2010). As expected, in free-living conditions, expression of the reporter fusion, assessed by measuring β-galactosidase activity, was upregulated approx. 15-fold in the presence of the NO donor spermine NONOate (25 μM) in the wt strain. By contrast, no significant upregulation of the reporter fusion was observed in an *nnrR* mutant, or in a wt strain carrying the empty vector pCZ962 (Fig. 2). Thus, this strain was used as a NO biosensor strain. The bacteria carrying the reporter fusion were used to inoculate *M. truncatula* plantlets, and expression of the fusion was assessed, 6 d post-inoculation, by X-Gal staining and microscopic analysis of the roots. Blue-stained bacteria entrapped in ‘shepherd's crooks’ of root hairs (Fig. 3) were detected on roots inoculated with the wt strain, whereas no staining was observed with the *nnrR* mutant or with the wt strain carrying the empty vector pCZ962 (data not shown). Also, no staining was detected when we used a *S. meliloti* strain overexpressing the flavohaemoglobin Hmp from plasmid pBBR-MCS5 (Meilhoc *et al.*, 2010). Together, these results confirm that NO is present in the infection pockets and infection threads, and indicate that bacteria respond to NO in infection pockets.

The DAF-2T fluorescence was also observed 4 dpi in the bumps of the root infection zone in the region of infection thread progression. To more precisely localize this NO production, we carried out confocal microscopic analysis on fresh root slices (100 μm) from the infection zone (Fig. 4). All the pictures showed the fluorescence in dividing cortical cells of the nodule primordia (Fig. 4a,b). At the same time, we also detected NO production in lateral root primordia (data not shown), as described in lateral root development in tomatoes (Correa-Aragunde *et al.*, 2004). As expected, this green fluorescence was significantly diminished by treating root slices with cPTIO (Fig. 4c,d). These results show that NO is present in the early stages of the symbiotic interaction.

### NO is necessary for efficient competitiveness of *S. meliloti*

To determine whether the NO detected at early steps of symbiosis is involved in the infection process, we used an approach to deplete the NO levels. For this, we first tested the ability of the *S. meliloti hmp*^*++*^ strain to compete with the wt strain for nodule occupancy. *Medicago truncatula* plants were coinoculated with a mixture (1 : 1) of the wt and *hmp*^++^ strain. To differentiate nodules occupied by each strain, the wt harboured plasmid pXLGD4, which displays a constitutive and strong β-gal activity. Nodules occupied by the wt and *hmp*^++^ strains could thus be numbered after addition of X-Gal by counting blue and white nodules, respectively. In control experiments, the wt strain harbouring pXLGD4 was mixed (1 : 1) with either the empty wt strain, or the wt strain carrying the empty vector pBBR-MCS5.

The competition experiment revealed a lower competitiveness of the *hmp*^++^ strain compared with the wt (31.6% vs 68.4% nodules occupancy, respectively; *P* < 0.05 according to Student's *t*-test) whereas in control experiments, all strains were as competitive as the wt (Table 2). Even when the competition experiment was done with a 1 : 3 mixture of the wt vs *hmp*^++^, the *hmp*^++^ strain still remained significantly less competitive (36.5%, *n* = 1). It has been shown that overexpression of *hmp* in bacteria can lead to elevated concentrations of superoxide and peroxide (Gilberthorpe *et al.*, 2007; McLean *et al.*, 2010). To rule out the possibility that such an effect is responsible for the lower competitiveness of the *hmp*^*++*^*strain*, the coinoculation of wt and *hmp*^++^ strains was repeated in the presence of the NO scavenger (cPTIO). In these conditions, the wt and *hmp*^++^ strains recovered a similar competitiveness. This confirmed that the low competitivity of the *hmp*^++^ strain was indeed the result of its greater ability to degrade NO. These results therefore indicate that NO production has a positive effect on the early steps of bacterial infection.

**Table 2:** Nodulation competitiveness of wild type (wt) and flavohaemoglobin (*hmp*^++^) over-expressing strain

Strain tested^a^	cPTIO treatment (1 mM)	Per cent of nodule occupancy (± SE)	Total number of nodules (number of experiments)
wt	−	51.3 (±2.3)	136 (2)
wt (pBBR-MCS5)	−	47.0 (±0.3)	121 (2)
*hmp*^*++*^	−	31.6 (±4.9)	481 (6)
*hmp*^*++*^	+	51.2 (±2.1)	183 (3)

a The strains indicated were tested in competition against the wt strain harbouring the plasmid pXLGD4 in a 1 : 1 ratio.

### NO is necessary for optimal nodule formation

**Fig. 5:**
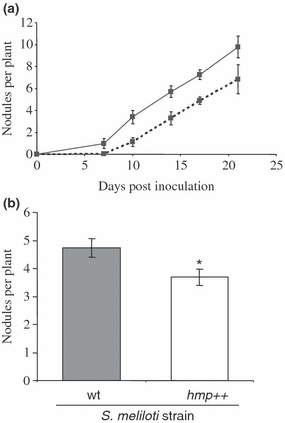
Nodulation phenotype on roots treated with cPTIO (2-(4-carboxyphenyl)-4,4,5,5-tetramethyl imidazoline-1-oxyl-3-oxide) or inoculated with *hmp*-overexpressing *Sinorhizobium meliloti* strain. (a) *Medicago truncatula* roots were either untreated (solid line) or treated with 1 mM cPTIO 2 h before, 2 h after and every 24 h during 4 d after inoculation with the wild-type (wt) *S. meliloti* strain (dotted line). Three independent series of 18 plants were tested for each condition. The average nodule number at each time-point was calculated for each experiment. The results shown are the mean (± mean deviation) of the three independent experiments. (b) Number of nodules formed by on *M. truncatula* roots 12 d post-inoculation by the wt (GMI11495, tinted bar) or the *hmp*-overexpressing strain (*hmp*^*++*^, open bar) *S. meliloti* strains. Three independent series of 20 plants were done. The results shown are the mean (± SE) of the three experiments. Asterisk indicates a statistically significant difference (Student's *t*-test, *P*<0.05).

To investigate the role of NO in the establishment of the symbiotic interaction, we first tested the effect of the NO scavenger cPTIO on the nodulation ability of *M. truncatula* plants inoculated by the *S. meliloti* wt strain. The plants grown in tubes were treated by addition of 1 mM cPTIO on the whole length of the root 2 h before and 2 h after inoculation, and then every 24 h for 4 d. Appearance of nodules was then monitored. The results show that the NO depletion caused by cPTIO treatment led to a significant delay (3–4 d) in nodule appearance (Fig. 5a). Similarly, plant inoculation with the *hmp*^*++*^ strain led to a slight but significant delay in nodule formation (Fig. 5b).

To corroborate these results, transgenic hairy roots overexpressing the bacterial *hmp* gene, encoding a flavohaemoglobin able to scavenge NO, were obtained. To avoid any effect on root development, the *hmp* gene was expressed under the control of a nodule specific promoter p*MtENOD20*. As described in the literature (Vernoud *et al.*, 1999), we observed the expression of the p*MtENOD20-GUS* construction in dividing inner cortical cells corresponding to the site of nodule primordium from the fifth day of nodule development (see the Supporting Information, Fig. S1). However, compared with the results of Vernoud *et al.* (1999), we did not observe glucuronidase (GUS) staining in associated root hairs displaying shepherd's crook curling. Detection of NO by fluorescent microscopy with DAF2-DA revealed a decrease of fluorescence on the Hmp-overexpressing transgenic nodule slices compared with the control transgenic nodules (Fig. 6a). Using a spectrofluorometric method, we estimated a 40% reduction of the NO production in hairy roots overexpressing the Hmp protein compared with the control hairy roots at 5 dpi (Fig. 6b) as well as at 13 dpi (data not shown). In addition, the overexpression of *hmp* under the control of this promoter significantly delayed the nodulation (Fig. 6c), with a 28% reduction in the number of nodules compared with the control transgenic roots. This result is in line with the results demonstrated with cPTIO treatment (Fig. 5a) or when plants were inoculated with the *hmp*^*++*^*S. meliloti* strain (Fig. 5b). These results show that decreasing the NO level results in a delay in the nodulation process.

### Depletion of NO affects transcript level of genes involved in nodulation process

To further investigate the involvement of NO in this process, we measured the expression levels of genes known to be associated with nodule development and potentially regulated by NO. We first analysed the expression of *MtCRE1*, a gene encoding a cytokinin receptor involved in the early symbiotic interaction (Gonzalez-Rizzo *et al.*, 2006) as it was shown to be NO regulated in *M. truncatula* (Ferrarini *et al.*, 2008). The qRT-PCR experiments performed either on inoculated plants treated with cPTIO (during 8 h) or on transgenic hairy roots overexpressing the Hmp protein (Fig. 7a,b) showed that NO depletion downregulates the expression of *MtCRE1*. This result therefore confirms that NO upregulates the expression of *MtCRE1*, and further points to the presence of NO during the early steps of nodule development.

We then measured the expression levels of several well-studied genes associated with various infection and nodule developmental stages: *MtRR4*, *MtNIN*, *MtN6, MtENOD40* and *MtCCS52A* (Pichon *et al.*, 1992; Charon *et al.*, 1997; Cebolla *et al.*, 1999; Vinardell *et al.*, 2003; Gonzalez-Rizzo *et al.*, 2006). Only one of them, *MtCCS52A*, displayed a lower expression level upon NO depletion by either cPTIO or Hmp overexpression (Fig. 7 and data not shown) suggesting that the gene expression could be induced by the NO production observed at this stage. During nodule organogenesis, the cell cycle-switching gene *MtCCS52A* triggers selected cells within the primordium to switch from mitotic cycles into endoreduplicating cycles (Cebolla *et al.*, 1999; Vinardell *et al.*, 2003). These data therefore strengthen the role of NO in nodule development.

## Discussion

In previous work, we showed that NO is present in late stages of nodule development (Baudouin *et al.*, 2006). The results presented here clearly show that NO is also present in the first steps of the *M. truncatula–S. meliloti* symbiosis, that is, in the root hair infection pockets as well as in the nodule primordium, clearly pointing to a role of this reactive nitrogen species in the early exchange of signals between the two partners. They are in agreement with previous reports of NO detection on *M. sativa* or *L. japonicus* roots a few hours after inoculation by the respective microsymbionts and further indicate that NO production occurs at different steps during the nodulation process (Shimoda *et al.*, 2005; Pii *et al.*, 2007; Nagata *et al.*, 2008). Furthermore, in each step the NO observed might have specific functions.

In order to evaluate the role of NO, a strategy could have been to diminish the NO production and analyse the consequences on the symbiotic steps. However, although numerous studies demonstrate the importance of NO in plants, NO synthesis pathway(s) are yet to be deciphered. Preliminary indications pointed out the possible implication of an NO synthase-like enzyme in *M. truncatula–S. meliloti* interaction (Baudouin *et al.*, 2006). However, the *MtNoa1* gene (the orthologue of *AtNoa1*) recently identified in *M. truncatula* is not involved in NO production (Pauly *et al.*, 2010). In addition, recent evidence demonstrates that both plant and bacterial nitrate reductase and the electron transfer chains are responsible for NO production in *M. truncatula–S. meliloti* nodules (Horchani *et al.*, 2011). However, the NO source(s) during the first steps of the interaction remain(s) to be identified. Therefore to reduce NO levels in both bacteria and plant cells, we first used an *S. meliloti* strain overexpressing a flavohaemoglobin (Hmp) (Meilhoc *et al.*, 2010), a well-characterized NO-scavenging enzyme. Second, to assess the role of NO during the nodule primordium development, we generated transgenic hairy roots of *M. truncatula* expressing the *S. meliloti* flavohaemoglobin *hmp* gene under the control of a nodule-specific promoter. As reported by Mishina *et al.* (2007) in *Arabidopsis*, this strategy was efficient in reducing the NO levels during the nodulation process and allowed us to observe effects both on bacterial infection and nodule development. We first observed a lower competitive ability of the *hmp*^*++*^ strain compared with the wild type. Moreover, expression of Hmp from either the plant or bacterial side, or the early addition of the NO scavenger cPTIO to plant roots, led to delayed nodulation. In agreement with these observations, Pii *et al.* (2007) described that early addition of cPTIO reduced the *M. truncatula* nodule number*.* Together, these results provide convincing evidence that NO is involved in the establishment of the *M. truncatula–S. meliloti* symbiosis, where it plays a positive role in the nodulation process.

What could be the function of NO in the nodulation process? It has been suggested that NO produced by bacterial pathogens could protect them against oxidative stress encountered during host infection (Gusarov & Nudler, 2005). Similarly, NO could thus be produced by rhizobia in the early stages of symbiosis in order to counteract the oxidative stress encountered during infection (Pauly *et al.*, 2006). Interestingly, it appears that NO is produced very early in the infection process, as soon as infection pockets are formed. Both cell-permeable NO-specific fluorescent probe and NO biosensor bacterial strain indicate that bacteria respond to NO in infection pockets. However, in this study, we cannot conclude what from the plant or the bacteria is involved in this NO synthesis. Furthermore, detection of NO along the infection threads but not within bacteria during the IT growth suggests that NO production at this step is mainly from the plant partner, and/or that bacterial system(s), including the flavohaemoglobin Hmp, prevent(s) NO accumulation inside bacteria (Meilhoc *et al.*, 2010). In the *Vibrio fischeri–Euprymna scolopes* symbiosis, host-derived NO has an important role during the initiation of symbiotic colonization where it is hypothesized to serve as a specificity determinant (Davidson *et al.*, 2004). It has been suggested that NO in this case could be a signal that prepares the microsymbiont for stronger NO stress occurring later in the symbiotic process (Wang *et al.*, 2010). Similarly, in the *Rhizobium*–legume symbiosis, early production of NO could serve to induce the expression of bacterial genes that could be necessary to adapt bacteria to NO encountered later on during symbiosis. The lower competitiveness of the *hmp*^++^ strain compared with the wt is an argument in favour of a role of this NO production during the infection process.

The question remains whether or not NO synthesis by the plant is directly induced by the microsymbiont. In *L. japonicus* and *M. sativa*, Nagata *et al.* (2008) observed a transient NO production on roots only when the plants were inoculated with their own specific symbiotic partners (*Mesorhizobium loti* and *S. meliloti*, respectively). Furthermore, this increase in NO was accompanied by the transient induction of the expression of a class 1 Hb-encoding gene which could be responsible for subsequent NO decrease. It was suggested that class 1 Hb might be involved in the development of symbiosis in *L. japonicus* (Shimoda *et al.*, 2009). The same authors also reported that the number of nodules formed on transgenic hairy roots overexpressing *LjHb1* was increased compared with those formed on untransformed hairy root from the same plant (Shimoda *et al.*, 2009). These results appear contradictory to the present work where we observed a reduced number of nodules per plant when depleting NO concentrations using either *hmp* overexpression or the NO scavenger cPTIO. This could be caused by: (1) the different models and approaches used, including the expression of *hmp* from different promoters (i.e. a nodule-specific promoter in our work vs the strong and constitutive p35S promoter in Shimoda's work); (2) a putative specific role played by the class I Hb to promote the initial infection process; and/or (3) the need for a set concentration range of NO for successful establishment of the symbiotic relationship.

The detection of NO in dividing cortical cells of the root, not yet invaded by the rhizobial cells within nodule primordia, shows that NO could also play a direct role in nodule organogenesis. Nitric oxide was also detected in lateral root primordia as previously described in tomato plants (Correa-Aragunde *et al.*, 2004), which suggests that it could have a similar function in these two different developmental processes. The role of NO in nodule development is underlined by the effect of NO depletion on the expression of genes known to be involved in this process. A first target appears to be *MtCRE1*, which has been shown already as an NO responsive gene (Ferrarini *et al.*, 2008). This gene encodes a cytokinin receptor, which regulates the early symbiotic interaction between *M. truncatula* and *S. meliloti*, by modulating both the progression of the infection and the formation of nodule primordia (Gonzalez-Rizzo *et al.*, 2006); its expression was localized in nodule primordia (Lohar *et al.*, 2006). It has been proposed that cytokinin, as a key differentiation signal for nodule organogenesis, might be a ‘secret agent’ of the symbiotic interaction (Frugier *et al.*, 2008). Thus, in regulating cytokinin perception, NO may control the nodulation process. Moreover, as NO has been put forward as an intermediate in cytokinin signalling in *Arabidopsis* (Tun *et al.*, 2008), an amplification loop may exist. The proposed event cascade involving the response regulator MtRR4 and the downstream MtNIN and MtENOD40 functions (Frugier *et al.*, 2008) was not affected by the cPTIO treatment, pointing to the existence of other signalling routes functioning downstream of MtCRE1 and controlling nodule development. The effect of the decrease in the NO content on the expression of the *MtCCS52A* genes may also contribute to explain the positive role of this signalling molecule on the nodulation process. MtCCS52A, which is involved in the transition of mitotic cycles to endoreduplication cycles, is indeed required for symbiotic cell differentiation in *M. truncatula* nodules (Vinardell *et al.*, 2003). These results are in line with a previous report on the NO-induced modulation of the expression of cell cycle regulatory genes during lateral root formation in tomato plants (Correa-Aragunde *et al.*, 2006).

In conclusion, the results presented here show that NO is required for an optimal establishment of the *M. truncatula*–*S. meliloti* symbiosis, and suggest that it could have functions in both bacterial infection and nodule development. Indeed, enhancing NO scavenging activities, both at the plant and microsymbiont levels, resulted in a delay in nodule formation and a weaker bacterial infectivity. They point to a potential link between cytokinin and NO signalling during the nodulation process. In addition, they emphasize a difference with the role of NO in pathogenic interactions, where it mainly activates plant defence reactions.

## Supporting Information

**Fig. S1** Histochemical localization of β-glucuronidase (GUS) activity throughout various stages of root nodulation in transgenic hairy roots of *Medicago truncatula* expressing the pMtENOD20–GUS fusion.

**Table S1** Primer sequences for cloning and quantitative reverse-transcription polymerase chain reaction (RT-PCR) analysis

## References

[b1] Baudouin E, Pieuchot L, Engler G, Pauly N, Puppo A (2006). Nitric oxide is formed in *Medicago truncatula–Sinorhizobium meliloti* functional nodules. Molecular Plant–Microbe Interactions.

[b2] Beligni MV, Lamattina L (2000). Nitric oxide stimulates seed germination and de-etiolation, and inhibits hypocotyl elongation, three light-inducible responses in plants. Planta.

[b3] Benedito VA, Torres-Jerez I, Murray JD, Andriankaja A, Allen S, Kakar K, Wandrey M, Verdier J, Zuber H, Ott T (2008). A gene expression atlas of the model legume *Medicago truncatula*. Plant Journal.

[b4] Besson-Bard A, Pugin A, Wendehenne D (2008). New insights into nitric oxide signaling in plants. Annual Review of Plant Biology.

[b5] Bethke PC, Libourel IG, Jones RL (2006). Nitric oxide reduces seed dormancy in Arabidopsis. Journal of Experimental Botany.

[b6] Boccara M, Mills CE, Zeier J, Anzi C, Lamb C, Poole RK, Delledonne M (2005). Flavohaemoglobin HmpX from *Erwinia chrysanthemi* confers nitrosative stress tolerance and affects the plant hypersensitive reaction by intercepting nitric oxide produced by the host. Plant Journal.

[b7] Boisson-Dernier A, Chabaud M, Garcia F, Becard G, Rosenberg C, Barker DG (2001). *Agrobacterium rhizogenes*-transformed roots of *Medicago truncatula* for the study of nitrogen-fixing and endomycorrhizal symbiotic associations. Molecular Plant–Microbe Interactions.

[b8] Bringhurst RM, Cardon ZG, Gage DJ (2001). Galactosides in the rhizosphere: utilization by *Sinorhizobium meliloti* and development of a biosensor. Proceedings of the National Academy of Sciences, USA.

[b9] de Bruijn FJ, Rossbach S, Bruand C, Parrish JR (2006). A highly conserved *Sinorhizobium meliloti* operon is induced microaerobically via the FixLJ system and by nitric oxide (NO) via NnrR. Environmental Microbiology.

[b10] Cebolla A, Vinardell JM, Kiss E, Olah B, Roudier F, Kondorosi A, Kondorosi E (1999). The mitotic inhibitor ccs52 is required for endoreduplication and ploidy-dependent cell enlargement in plants. EMBO Journal.

[b11] Charon C, Johansson C, Kondorosi E, Kondorosi A, Crespi M (1997). enod40 induces dedifferentiation and division of root cortical cells in legumes. Proceedings of the National Academy of Sciences, USA.

[b12] Correa-Aragunde N, Graziano M, Chevalier C, Lamattina L (2006). Nitric oxide modulates the expression of cell cycle regulatory genes during lateral root formation in tomato. Journal of Experimental Botany.

[b13] Correa-Aragunde N, Graziano M, Lamattina L (2004). Nitric oxide plays a central role in determining lateral root development in tomato. Planta.

[b14] Cueto M, Hernandez-Perera O, Martin R, Bentura ML, Rodrigo J, Lamas S, Golvano MP (1996). Presence of nitric oxide synthase activity in roots and nodules of *Lupinus albus*. FEBS Letters.

[b15] Davidson SK, Koropatnick TA, Kossmehl R, Sycuro L, McFall-Ngai MJ (2004). NO means ‘yes’ in the squid–vibrio symbiosis: nitric oxide (NO) during the initial stages of a beneficial association. Cellular Microbiology.

[b16] Delledonne M (2005). NO news is good news for plants. Current Opinion in Plant Biology.

[b17] Delledonne M, Xia Y, Dixon RA, Lamb C (1998). Nitric oxide functions as a signal in plant disease. Nature.

[b18] Ditta G, Schmidhauser T, Yakobson E, Lu P, Liang XW, Finlay DR, Guiney D, Helinski DR (1985). Plasmids related to the broad host range vector, pRK290, useful for gene cloning and for monitoring gene expression. Plasmid.

[b19] Durner J, Wendehenne D, Klessig DF (1998). Defense gene induction in tobacco by nitric oxide, cyclic GMP, and cyclic ADP-ribose. Proceedings of the National Academy of Sciences, USA.

[b20] Ferrarini A, De Stefano M, Baudouin E, Pucciariello C, Polverari A, Puppo A, Delledonne M (2008). Expression of *Medicago truncatula* genes responsive to nitric oxide in pathogenic and symbiotic conditions. Molecular Plant–Microbe Interactions.

[b21] Figurski DH, Helinski DR (1979). Replication of an origin-containing derivative of plasmid RK2 dependent on a plasmid function provided *in trans*. Proceedings of the National Academy of Sciences, USA.

[b22] Fournier J, Timmers AC, Sieberer BJ, Jauneau A, Chabaud M, Barker DG (2008). Mechanism of infection thread elongation in root hairs of *Medicago truncatula* and dynamic interplay with associated rhizobial colonization. Plant Physiology.

[b23] Frugier F, Kosuta S, Murray JD, Crespi M, Szczyglowski K (2008). Cytokinin: secret agent of symbiosis. Trends in Plant Science.

[b24] Gardner PR, Gardner AM, Martin LA, Salzman AL (1998). Nitric oxide dioxygenase: an enzymic function for flavohemoglobin. Proceedings of the National Academy of Sciences, USA.

[b25] Gilberthorpe NJ, Lee ME, Stevanin TM, Read RC, Poole RK (2007). NsrR: a key regulator circumventing *Salmonella enterica* serovar *typhimurium* oxidative and nitrosative stress *in vitro* and in IFN-gamma-stimulated J774.2 macrophages. Microbiology.

[b26] Gonzalez-Rizzo S, Crespi M, Frugier F (2006). The *Medicago truncatula* CRE1 cytokinin receptor regulates lateral root development and early symbiotic interaction with *Sinorhizobium meliloti*. Plant Cell.

[b27] Grun S, Lindermayr C, Sell S, Durner J (2006). Nitric oxide and gene regulation in plants. Journal of Experimental Botany.

[b28] Gusarov I, Nudler E (2005). NO-mediated cytoprotection: instant adaptation to oxidative stress in bacteria. Proceedings of the National Academy of Sciences, USA.

[b29] He Y, Tang RH, Hao Y, Stevens RD, Cook CW, Ahn SM, Jing L, Yang Z, Chen L, Guo F (2004). Nitric oxide represses the Arabidopsis floral transition. Science.

[b30] Horchani F, Prevot M, Boscari A, Evangelisti E, Meilhoc E, Bruand C, Raymond P, Boncompagni E, Aschi-Smiti S, Puppo A (2011). Both plant and bacterial nitrate reductases contribute to nitric oxide production in *Medicago truncatula* nitrogen-fixing nodules. Plant Physiology.

[b31] Igamberdiev AU, Hill RD (2004). Nitrate, NO and haemoglobin in plant adaptation to hypoxia: an alternative to classic fermentation pathways. Journal of Experimental Botany.

[b32] Ignarro LJ (2000). Nitric oxide. Biology and pathobiology.

[b33] Kojima H, Nakatsubo N, Kikuchi K, Kawahara S, Kirino Y, Nagoshi H, Hirata Y, Nagano T (1998). Detection and imaging of nitric oxide with novel fluorescent indicators: diaminofluoresceins. Analytical Chemistry.

[b34] Kovach ME, Elzer PH, Hill DS, Robertson GT, Farris MA, Roop RM, Peterson KM (1995). Four new derivatives of the broad-host-range cloning vector pBBR1MCS, carrying different antibiotic-resistance cassettes. Gene.

[b35] Lohar DP, Sharopova N, Endre G, Penuela S, Samac D, Town C, Silverstein KA, VandenBosch KA (2006). Transcript analysis of early nodulation events in *Medicago truncatula*. Plant Physiology.

[b36] Long SR (2001). Genes and signals in the *Rhizobium*–legume symbiosis. Plant Physiology.

[b37] Mathieu C, Moreau S, Frendo P, Puppo A, Davies MJ (1998). Direct detection of radicals in intact soybean nodules: presence of nitric oxide-leghemoglobin complexes. Free Radical Biology and Medicine.

[b38] McLean S, Bowman LA, Poole RK (2010). Peroxynitrite stress is exacerbated by flavohaemoglobin-derived oxidative stress in *Salmonella typhimurium* and is relieved by nitric oxide. Microbiology.

[b39] Meakin GE, Bueno E, Jepson B, Bedmar EJ, Richardson DJ, Delgado MJ (2007). The contribution of bacteroidal nitrate and nitrite reduction to the formation of nitrosylleghaemoglobin complexes in soybean root nodules. Microbiology.

[b40] Meilhoc E, Cam Y, Skapski A, Bruand C (2010). The response to nitric oxide of the nitrogen-fixing symbiont *Sinorhizobium meliloti*. Molecular Plant–Microbe Interactions.

[b41] Miller JH (1972). Experiments in molecular genetics.

[b42] Mishina TE, Lamb C, Zeier J (2007). Expression of a nitric oxide degrading enzyme induces a senescence programme in Arabidopsis. Plant, Cell & Environment.

[b43] Moreau M, Lee GI, Wang Y, Crane BR, Klessig DF (2008). AtNOS/AtNOA1 is a functional *Arabidopsis thaliana* cGTPase and not a nitric-oxide synthase. The Journal of Biological Chemistry.

[b44] Mur LAJ, Carver TLW, Prats E (2006). NO way to live; the various roles of nitric oxide in plant–pathogen interactions. Journal of Experimental Botany.

[b45] Nagata M, Murakami E, Shimoda Y, Shimoda-Sasakura F, Kucho K, Suzuki A, Abe M, Higashi S, Uchiumi T (2008). Expression of a class 1 hemoglobin gene and production of nitric oxide in response to symbiotic and pathogenic bacteria in *Lotus japonicus*. Molecular Plant–Microbe Interactions.

[b46] Neill SJ, Desikan R, Clarke A, Hancock JT (2002). Nitric oxide is a novel component of abscisic acid signaling in stomatal guard cells. Plant Physiology.

[b47] Neill SJ, Desikan R, Hancock JT (2003). Nitric oxide signalling in plants. New Phytologist.

[b48] Oke V, Long SR (1999). Bacterial genes induced within the nodule during the *Rhizobium*–legume symbiosis. Molecular Microbiology.

[b49] Oldroyd GE, Downie JA (2008). Coordinating nodule morphogenesis with rhizobial infection in legumes. Annual Review of Plant Biology.

[b50] Pauly N, Ferrari C, Andrio E, Marino D, Piardi S, Brouquisse R, Baudouin E, Puppo A (2010). MtNOA1/RIF1 modulates *Medicago truncatula–Sinorhizobium meliloti* nodule development without affecting its nitric oxide content. Journal of Experimental Botany.

[b51] Pauly N, Pucciariello C, Mandon K, Innocenti G, Jamet A, Baudouin E, Herouart D, Frendo P, Puppo A (2006). Reactive oxygen and nitrogen species and glutathione: key players in the legume–*Rhizobium* symbiosis. Journal of Experimental Botany.

[b52] Pichon M, Journet EP, Dedieu A, de Billy F, Truchet G, Barker DG (1992). *Rhizobium meliloti* elicits transient expression of the early nodulin gene ENOD12 in the differentiating root epidermis of transgenic alfalfa. Plant Cell.

[b53] Pii Y, Crimi M, Cremonese G, Spena A, Pandolfini T (2007). Auxin and nitric oxide control indeterminate nodule formation. BMC Plant Biology.

[b54] Pobigaylo N, Szymczak S, Nattkemper TW, Becker A (2008). Identification of genes relevant to symbiosis and competitiveness in *Sinorhizobium meliloti* using signature-tagged mutants. Molecular Plant–Microbe Interactions.

[b55] Poole RK, Hughes MN (2000). New functions for the ancient globin family: bacterial responses to nitric oxide and nitrosative stress. Molecular Microbiology.

[b56] Quandt HJ, Puhler A, Broer I (1993). Transgenic root-nodules of *Vicia hirsuta*– a fast and efficient system for the study of gene-expression in indeterminate-type nodules. Molecular Plant–Microbe Interactions.

[b57] del Rio LA, Corpas FJ, Barroso JB (2004). Nitric oxide and nitric oxide synthase activity in plants. Phytochemistry.

[b58] Rosenberg C, Boistard P, Dénarié J, Casse-Delbart F (1981). Genes controlling early and late functions in symbiosis are located on a megaplasmid in *Rhizobium meliloti*. Molecular and General Genetics.

[b59] Sambrook J, Fritsch EF, Maniatis T (1989). Molecular cloning: a laboratory manual.

[b60] Shimoda Y, Nagata M, Suzuki A, Abe M, Sato S, Kato T, Tabata S, Higashi S, Uchiumi T (2005). Symbiotic *Rhizobium* and nitric oxide induce gene expression of non-symbiotic hemoglobin in *Lotus japonicus*. Plant and Cell Physiology.

[b61] Shimoda Y, Shimoda-Sasakura F, Kucho K, Kanamori N, Nagata M, Suzuki A, Abe M, Higashi S, Uchiumi T (2009). Overexpression of class 1 plant hemoglobin genes enhances symbiotic nitrogen fixation activity between *Mesorhizobium loti* and *Lotus japonicus*. Plant Journal.

[b62] Stevanin TM, Poole RK, Demoncheaux EA, Read RC (2002). Flavohemoglobin Hmp protects *Salmonella enterica* serovar *typhimurium* from nitric oxide-related killing by human macrophages. Infection and Immunity.

[b63] Svensson L, Poljakovic M, Save S, Gilberthorpe N, Schon T, Strid S, Corker H, Poole RK, Persson K (2010). Role of flavohemoglobin in combating nitrosative stress in uropathogenic *Escherichia coli*– implications for urinary tract infection. Microbial Pathogenesis.

[b64] Tun NN, Livaja M, Kieber JJ, Scherer GF (2008). Zeatin-induced nitric oxide (NO) biosynthesis in *Arabidopsis thaliana* mutants of NO biosynthesis and of two-component signaling genes. New Phytologist.

[b65] Van de Velde W, Guerra JC, De Keyser A, De Rycke R, Rombauts S, Maunoury N, Mergaert P, Kondorosi E, Holsters M, Goormachtig S (2006). Aging in legume symbiosis. A molecular view on nodule senescence in *Medicago truncatula*. Plant Physiology.

[b66] Vandesompele J, De Preter K, Pattyn F, Poppe B, Van Roy N, De Paepe A, Speleman F (2002). Accurate normalization of real-time quantitative RT-PCR data by geometric averaging of multiple internal control genes. Genome Biology.

[b67] Vernoud V, Journet EP, Barker DG (1999). MtENOD20, a Nod factor-inducible molecular marker for root cortical cell activation. Molecular Plant–Microbe Interactions.

[b68] Vinardell JM, Fedorova E, Cebolla A, Kevei Z, Horvath G, Kelemen Z, Tarayre S, Roudier F, Mergaert P, Kondorosi A (2003). Endoreduplication mediated by the anaphase-promoting complex activator CCS52A is required for symbiotic cell differentiation in *Medicago truncatula* nodules. Plant Cell.

[b69] Wang Y, Dunn AK, Wilneff J, McFall-Ngai MJ, Spiro S, Ruby EG (2010). *Vibrio fischeri* flavohemoglobin protects against nitric oxide during initiation of the squid–*Vibrio* symbiosis. Molecular Microbiology.

