# Supplementary material for: Nitric oxide is required for an optimal establishment of the Medicago truncatula–Sinorhizobium meliloti symbiosis
Source: New Phytol. 2011 Jul;191(2):405–17. doi: 10.1111/j.1469-8137.2011.03693.x (PMC3147055; doi:10.1111/j.1469-8137.2011.03693.x)
Supplement: Supplementary file 1 [file nph0191-0405-SD1.doc]

| *Primers for plasmid constructions* | | | | |  |  | | |
| --- | --- | --- | --- | --- | --- | --- | --- | --- |
| ***name*** | **forward primer** | | | | ***name*** | **reverse primer** | | |
| ***DsRedX*** | CAGTCTCGAGCCACCATGGCGCGCTCCTCCAAGAA | | | | ***DsRedS*** | CGAGAGCTCCCCGATCTAGTAACATAG | | |
| ***a74F*** | GGGGACAACTTTGTATAGAAAAGTTGAGGATGTGACGGTGAATGAT | | | | ***a74R*** | GGGGACTGCTTTTTTGTACAAACTTGAGTGAAAGATTAATTAGAATTGAGT | | |
| ***a76F*** | AAAAAGCAGGCTTCACCATGCTCACTCAGAAGACCAA | | | | ***a76R*** | AGAAAGCTGGGTGTTACTAGATCACTCGGCAAACA | | |
| ***OCB744*** | AACTGCAGGACCCCCTGCTGGTA | | | | ***OCB745*** | GCTCTAGAGCGCACCGCGCTTCGTC | | |
| *Primers for RT-qPCR analysis* | | | |  | | |  |  |
| ***name*** | | **forward primer** | **reverse primer** | | | | **Affymetrix probeset** | **Reference** |
| ***Mtc27*** | | TTTATGGCGATCCTTATCTCACCAC | ACAATGGGCAGTATTTGACACTCCA | | | | Mtr.27459.1.S1_s_at | This work |
| ***a38*** | | CATGCTTCAACAGAGGGAGTCACTAA | AGATCATTCCTCCACCGAGAAGAAG | | | | Mtr.10324.1.S1_at | This work |
| ***a39*** | | AAGAAGCCAGAGACAACCAACAGG | CGTGGCATTGCATTAGTTACCACA | | | | Mtr.31250.1.S1_at | This work |
| ***MtCRE1*** | | CTCTTGCCATCCTTGTTTCAA | GTGCATAGGCCACTCCACTAA | | | | Mtr.45840.1.S1_at | This work |
| ***MtCCS52A*** | | TTGGCTGTTGGTTTGGGTAAC | GTTCCAACAGCAAGATGAGTA | | | | Mtr.12852.1.S1_at | Kuppusamy *et al.,* 2004 |
| ***MtN6*** | | GGCTTCTCTGTACTCCCAGGTT | AAACAACTTCGCGAGCATTC | | | | Mtr.43850.1.S1_at | Teillet *et al.*, 2008 |
| ***MtENOD40*** | | TCTCCAAAGGATCAGAAG | GCAGAAACTGAAACAAGAAC | | | | Mtr.12359.1.S1_at | Sauviac *et al.*, 2005 |
| ***MtRR4*** | | ATGCTTTTGTTCCGGGTTTA | CTGCACCTTCCTCCAAACAT | | | | Mtr.9656.1.S1_at | Gonzalez-Rizzo *et al.*, 2006 |
| ***MtNIN*** | | GGAAGATTGAGAGGGGAAGCTT | GCAATGTGGGGATTTAGAGATT | | | | Mtr.28094.1.S1_at | Gonzalez-Rizzo *et al.*, 2006 |

**Supporting Information Table S1, Fig.S1**

**Table S1** Primer sequences for cloning and quantitative RT-PCR analysis

**Fig. S1** Histochemical localization of β-glururonidase (GUS) activity throughout various stages of root nodulation in transgenic hairy roots of *M. truncatula* expressing the pMtENOD20-GUS fusion. (a) Staining of nodules primordia 4 d post inoculation. (b) Staining of 11-d-old nodule slice. Bars, 100 µm.

(a)

(b)
